# Proposed Remedy for Spina Bifida

**Published:** 1840

**Authors:** 


### PROPOSED REMEDY FOR SPINA BIFIDA.

The general failure of the existing methods of treating Spina bifida, may justify the suggestion of a new and different treatment. We beg leave to propose to the consideration of our brethren the following:

Let the cyst be emptied by a puncture, and then fill it with blood from the brachial vein, or temporal artery of the little patient. If practicable, cause the blood to flow from the vein or artery, into the sac, without cooling or being exposed to the air. If a small portion of the serum naturally contained in the cyst be left in its neck, none of the injected blood will enter the spinal theca. The mass of it will coagulate, its serum and coloring matter will be absorbed, or may be drawn off by a puncture, and the coagulated or concrete fibrin left behind. As fibrin thus situated, is susceptible of organization and adhesion to the surrounding tissues, it may be expected to undergo those changes in the cyst; and, according to its quantity, contribute to fill it up with solid matter. A second or third injection might be practised if necessary. In this way, would not a solid tumour take the place of one composed of serum? Might not the spinal orifice be thus closed up? And would not the tumour be gradually absorbed, at least in part? On principle, we do not see why this method should not be both safe and successful. It may be predicted, that it would excite a dangerous inflammation. But have we not many examples of extravasation of blood, into serous and cellular cavities, even into the substance of the brain, without any dangerous irritation of the surrounding parts? Undoubtedly it would be pernicious to inject the sac, with any dead and foreign fluid, capable of hardening, but a mass of organizable fibrin, must not, in its effects upon the serous lining of the sac, be compared with a lump of unorganizable matter. Should any of our readers meet with a case of spina bifida, among our domestic animals, we hope they will give this method a trial; and should they have a case of the same kind, in a child, which had resisted the usual treatment, and was likely to prove fatal, why should not this method be employed?

The blood might be taken from another young subject, than the patient. It should be caused to flow through the inverted intestine of a fowl; or be received into the inverted bladder of a recently killed animal, immersed in warm water. The less its contact with dead matter, the greater the probability of its becoming organized in the sac.

We hope to hear that some of our readers have tried this experiment. D.

